# Volcano-type catalytic activity driven by graphitization-dependent electron transfer in Pt@C core–shell catalysts

**DOI:** 10.3389/fchem.2026.1892260

**Published:** 2026-07-22

**Authors:** Xing Li, Xiaonian Li

**Affiliations:** State Key Laboratory Breeding Base of Green Chemistry Synthesis Technology, Industrial Catalysis Institute of Zhejiang University of Technology, Hangzhou, China

**Keywords:** graphitization degree, interfacial electron transfer, Pt@C core-shell catalysts, selective hydrogenation of p-chloronitrobenzene, volcano-type activity

## Abstract

Carbon-encapsulated metal catalysts can have a high stability. The catalytic activation mechanism of the inert carbon shell requires further investigation. We prepared platinum-core carbon-shell catalysts on activated carbon supports. The graphitization degree of the carbon layer was tuned through pyrolysis temperature control. The catalysts were tested in the selective hydrogenation of p-chloronitrobenzene. The intact carbon shell physically isolates the metal core. All coated samples achieved a target product selectivity above 99%. The catalytic activity exhibited a distinct volcano trend. The sample treated at 800 °C delivered the highest activity. Experimental and theoretical analyses revealed an interfacial electron transfer process. The metal core acts as an electron pump to enrich the carbon surface. The optimal activity originates from a balance between electronic and geometric effects. The electron-rich surface lowers the hydrogen dissociation barrier. The preserved carbon defects serve as necessary adsorption sites. The core-shell structure suppressed metal sintering and the catalyst maintained good stability over 10 reaction cycles. This study provides a basic understanding for the design of highly active carbon-functionalized interfaces.

## 1 Introduction

Selective hydrogenation of chloronitrobenzene (CNB) to chloroaniline (CAN) is an important reaction to produce several fine chemicals, pharmaceuticals, and dyes (Da et al., 2023; Jaf et al., 2018; Zhu et al., 2020a). A long-standing challenge in this reaction is to achieve high selectivity. Conventional noble metal catalysts, e.g., Pt and Pd, have an intrinsic tendency to promote undesired hydrodechlorination (Lu et al., 2019; Sun et al., 2023; Wang et al., 2022; Xiao et al., 2023). This is the major reason for the trade-off between catalytic activity and selectivity in this transformation (Guo et al., 2022), which heavily constrains the efficiency of conventional catalytic systems.

To overcome this barrier, many attempts have been made to regulate the catalytic behaviour of metal surfaces via several approaches, such as geometric site blocking and electronic structure modulation by surface modification (Li et al., 2018; Liu et al., 2026; Tan et al., 2026; Yun et al., 2026) or heteroatom doping (Li J. et al., 2022; Wang et al., 2022; Yao et al., 2024a). Although these strategies can partly suppress the unwanted side reactions, they fundamentally depend on the metal surface as the catalytic interface (Liang et al., 2025; Zhu et al., 2020b). Hence, the intrinsic limitations arising from the metal-catalysed C–Cl bond activation cannot be completely solved (Zhu et al., 2020b).

Encapsulating metal nanoparticles within carbon shells, namely, M@C core-shell structures, offers a promising alternative strategy (He et al., 2025; Li et al., 2020; Li S. et al., 2022; Martinez et al., 2018; Shen et al., 2026). Traditionally, the carbon shell is regarded as a physical barrier that protects the metal core from sintering and deactivation (Chen et al., 2025; Choi et al., 2023; Jiang et al., 2024). However, an intriguing question arises: can the carbon shell itself serve as an active catalytic interface? We hypothesize that the encapsulated metal core can act as an “electron pump”, injecting electrons into the surrounding carbon shell via interfacial charge transfer (Fonseca et al., 2026; Lin et al., 2023; Thanh, 2026), thereby activating the otherwise inert carbon surface (Alabi et al., 2025; Luengo et al., 2026; Zhan et al., 2024). In this context, the graphitization degree of the carbon shell is expected to play a crucial role by governing both electron transport and the density of surface defect sites (Fan et al., 2016; Flores-Lasluisa et al., 2024; Goldie and Coleman, 2023; Krishnan et al., 2025; Lindner et al., 2022; Lv et al., 2023; Wu et al., 2012).

In this work, a series of Pt@C/AC core-shell catalysts with systematically tunable graphitization degrees were synthesized via controlled pyrolysis. A volcano-type relationship between catalytic activity and graphitization degree was identified, while maintaining near-complete selectivity (>99%) toward CAN. Combined experimental characterizations (Raman, XPS, UPS, H_2_-TPD) and density functional theory (DFT) calculations reveal that the optimal performance originates from a delicate balance between enhanced interfacial electron transfer and the availability of defect sites for H_2_ activation. This study provides new insights into the design of carbon-based catalytic interfaces beyond conventional metal-centered paradigms.

## 2 Experimental

### 2.1 Materials and reagents

All commercial chemicals were of analytical grade and used as received without any further purification. Activated carbon (AC) was used as the catalyst support. Purchased from Sinopharm Chemical Reagent Co., Ltd. Chloroplatinic acid hydrate (H_2_PtCl_6_·xH_2_O, AR) and p-chloronitrobenzene (p-CNB, 99%) were purchased from Shanghai Macklin Biochemical Co., Ltd. And Shanghai Aladdin Biochemical Technology Co., Ltd., respectively. D-glucose (AR), methanol (AR), and absolute ethanol (AR) were supplied by Sinopharm Chemical Reagent Co., Ltd. High-purity hydrogen (H_2_>99.9%), nitrogen (N_2_ >99.9%), and a reducing gas mixture (5% H_2_/Ar) were provided by Hangzhou Jingong Special Gas Co., Ltd. Ultrapure water (18.2 MΩ cm) produced by a laboratory water purification system was used in all experiments.

### 2.2 Catalyst preparation

#### 2.2.1 Synthesis of the uncoated precursor

Activated carbon was calcined in air at 300 °C for 2 h. Chloroplatinic acid hydrate was dissolved in ultrapure water. 2 g of the treated carbon was impregnated with the platinum solution. The theoretical platinum loading was set to 3 wt%. The wet sample was sealed and aged at room temperature for 12 h. The mixture was then dried at 80 °C for 12 h. The dried solid was heated to 350 °C at a rate of 5 °C/min under a nitrogen flow. A reducing gas mixture containing 5% hydrogen in nitrogen was introduced into the furnace. The reduction proceeded at 350 °C for 3 h. The sample was cooled to room temperature under nitrogen and labeled as Pt/AC.

#### 2.2.2 Synthesis of the core-shell catalysts

0.5 g of the Pt/AC precursor was dispersed in 80 mL of ultrapure water. The suspension was sonicated for 30 min. A certain amount of D-glucose was added to the liquid under continuous magnetic stirring. The homogeneous mixture was transferred into a 100 mL Teflon-lined autoclave. The hydrothermal reaction was maintained at 180 °C for 12 h. The solid product was collected by filtration after natural cooling. The solid was washed with ultrapure water and ethanol multiple times until reaching a neutral state. The washed sample was vacuum-dried at 80 °C for 12 h. The dried powder was divided into four equal portions. The samples were heated to target temperatures of 600, 700, 800, and 900 °C in a tube furnace. The heating rate was 10 °C/min under a nitrogen flow. The target temperature was kept constant for 2 h. The solid products were cooled to room temperature under nitrogen. The obtained samples were labeled as Pt@C/AC-T. The letter T represents the specific pyrolysis temperature. The four final catalysts were named Pt@C/AC-600, Pt@C/AC-700, Pt@C/AC-800, and Pt@C/AC-900. All prepared catalysts were stored in a sealed desiccator.

### 2.3 Catalyst characterization

The crystal structures were analyzed using a PANalytical X’Pert PRO X-ray diffractometer with a Cu Kα radiation source. The scanning angle ranged from 10 to 80°. The microscopic morphology and particle size were observed via a Tecnai G2 F30 transmission electron microscope. The average metal particle sizes and size distributions were determined by statistically measuring randomly selected nanoparticles from the TEM images using the Nano Measurer software. The operating voltage was set to 300 kV. Nitrogen adsorption-desorption isotherms were measured at 77 K using a Micromeritics ASAP 2020 analyzer. All samples were degassed at 200 °C for 6 h under vacuum before testing. The specific surface areas were calculated through the Brunauer–Emmett–Teller method. The pore volumes and distributions were determined by the Barrett Joyner Halenda model. Raman spectra were recorded on a Horiba LabRAM HR Evolution spectrometer. A 532 nm laser served as the excitation source. This technique evaluated the structural defects and graphitization degrees of the carbon materials. Hydrogen temperature programmed reduction was performed on a Micromeritics AutoChem II 2920 analyzer. The samples were heated from 50 °C to 800 °C at a rate of 10 °C/min in a hydrogen and argon gas mixture. Hydrogen temperature programmed desorption was conducted on a Hiden QGA system equipped with a mass spectrometer. The samples were saturated with hydrogen at 40 °C and subsequently heated to 600 °C under an argon flow. X ray photoelectron spectroscopy was performed on a Thermo Scientific ESCALAB 250Xi system equipped with a monochromatic Al Kα source. The binding energies were calibrated using the surface adventitious carbon 1s peak at 284.8 eV. Ultraviolet photoelectron spectroscopy was conducted on the exact same instrument. A helium I gas discharge lamp providing 21.22 eV excitation energy was utilized to measure the catalyst work functions.

### 2.4 Catalytic performance evaluation

The liquid phase selective hydrogenation of p-chloronitrobenzene was performed in a stainless-steel autoclave. A certain amount of catalyst and reactant and 50 mL of methanol were placed into the reactor. The system was sealed and flushed with pure nitrogen gas six times to remove residual air. The reactor was then purged with pure hydrogen gas six times. The mixture was heated to 80 °C under mild stirring. Pure hydrogen gas was introduced to reach a constant pressure of 1.0 MPa. The stirring speed was raised to 1,000 rpm. This high stirring rate eliminated the external mass transfer resistance. The reaction started at this moment.

A temperature controller maintained the system at 80 °C. The hydrogen pressure was kept constant at 1.0 MPa through continuous gas supplementation. The reaction ended when the hydrogen consumption completely stopped. After cooling the reactor to room temperature, the pressure was released. The solid catalyst was separated from the liquid phase by a syringe filter.

The liquid-phase products were analyzed by an Agilent gas chromatograph. It was equipped with a FID detector and HP-5 capillary column. Qualitative analysis was performed by comparing the retention time with standard samples. The substrate conversion and product selectivity were calculated according to the normalized peak areas.

### 2.5 Kinetic evaluation

For the purpose of evaluating intrinsic catalytic activity, kinetic experiments were applied. Samples were taken at specific time intervals during the initial stage of the reaction. The total conversion was strictly controlled below 20%. The concentration of reactant was measured as a function of the reaction time. The initial reaction rate (r_0_) was obtained by linearly fitting the initial data points. The linear correlation coefficient was strictly greater than 0.99 for all calculations.

The turnover frequency (TOF) values were calculated to compare the specific activities. The apparent turnover frequency was adopted for all carbon-coated catalysts. TOF is calculated according to the following formula:

$$\text{TOF}=\left(r_{0}\times V\right)\;/\;n_{\text{Pt}‐\text{total}}$$

V: The total volume of the reaction liquid

n_Pt-total_: The total moles of platinum present in the catalyst sample.

A different normalization standard was applied to the uncoated reference catalyst. Its true turnover frequency was calculated based on the actual number of exposed surface active sites. This surface active site value was directly derived from the carbon monoxide pulse chemisorption measurement.

### 2.6 DFT calculations

Density functional theory calculations were performed using the CASTEP module in Materials Studio software. The Perdew–Burke–Ernzerhof generalized gradient approximation functional was applied to describe the exchange-correlation energy. Ultrasoft pseudopotentials were used to treat the core-valence electron interactions. Van der Waals interactions were corrected using the Grimme dispersion method. The plane-wave cutoff energy was set to 400 eV. The energy convergence criterion was 0.00001 eV/Å. The maximum force tolerance was 0.03 eV per angstrom. The Brillouin zone was sampled with a 3✕3✕1 Monkhorst-Pack k-point mesh. Transition states were located using the linear synchronous transit and quadratic synchronous transit methods. Bader charge analysis was used to calculate the atomic charge populations and quantify the electron transfer. The adsorption energy was calculated by subtracting the energies of the clean surface slab and the isolated gas molecule from the total energy of the adsorbed system. A more negative adsorption energy value indicates a stronger binding interaction between the molecule and the catalyst surface.

## 3 Results and discussion

The catalytic performance of the synthesized samples was evaluated in the liquid phase selective hydrogenation of p-chloronitrobenzene. Table 1 displays the catalytic performance. The uncoated Pt/AC catalyst exhibited high reactivity but poor selectivity. It achieved a remarkable turnover frequency of 28247 h^-1^. The selectivity toward the target product p-chloroaniline was only 71.8%. A large fraction of aniline byproduct was generated during the reaction. The exposed platinum metal sites possess a strong capability to cleave the carbon chlorine bonds.

**TABLE 1:** Catalytic performance data of Pt/AC and Pt@C/AC-T series catalysts.

Catalyst	p-CNB conversion (%)	Selectivity (%)		TOF (h^-1^)
		p-CAN	Aniline	
Pt/AC	100	71.8	28.2	28247
Pt@C/AC-600	100	99.2	0.8	1931
Pt@C/AC-700	100	99.6	0.4	2,684
Pt@C/AC-800	100	99.8	0.2	3,511
Pt@C/AC-900	100	99.7	0.3	1779

Reaction conditions: 0.05 g catalyst, 0.5 g p-CNB, 50 mL methanol, P = 1 MPa, r = 1,000 rpm, T = 80 °C.

In sharp contrast to the uncoated Pt/AC, which only showed 71.8% selectivity, the application of the carbon shell fundamentally improved the product distribution. All Pt@C/AC series catalysts delivered a p-chloroaniline selectivity above 99%. The hydrodechlorination side reaction was almost entirely suppressed. This near-complete selectivity is highly impressive compared with conventional unmodified noble-metal catalysts reported in the literature (Xiao et al., 2023; Zhang et al., 2020; Zhou et al., 2022), which typically suffer from severe hydrodechlorination and exhibit much lower target product selectivities.

**FIGURE 1:**
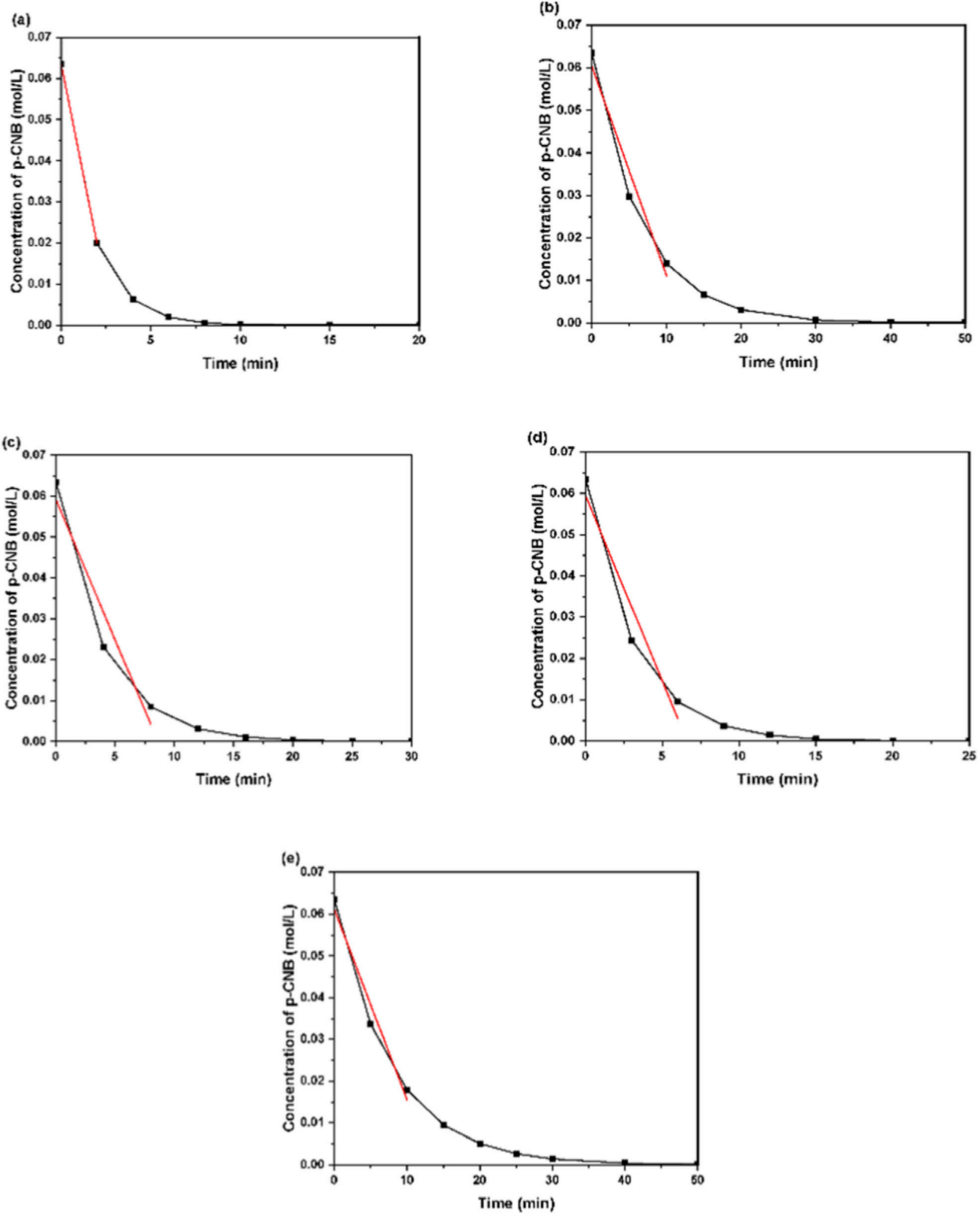
Reaction kinetics curve of catalyst: **(a)** Pt/AC, **(b)** Pt@C/AC-600, **(c)** Pt@C/AC-700, **(d)** Pt@C/AC-800, **(e)** Pt@C/AC-900.

Reaction kinetics were evaluated to compare the intrinsic activities of the coated catalysts. Initial reaction rates were extracted from the linear portion of the kinetic curves at low conversion levels. Apparent turnover frequency values were then normalized by the total moles of platinum in the system. The results are shown in Figure 1 and Table 2. The catalytic activity exhibited a distinct volcano trend as a function of the pyrolysis temperature. The turnover frequency of the Pt@C/AC-600 sample was 1931 h^-1^. This value increased to 2,684 h^-1^ when the pyrolysis temperature was raised to 700 °C. The maximum activity was observed on the Pt@C/AC-800 catalyst with a turnover frequency of 3,511 h^-1^. Raising the pyrolysis temperature further to 900 °C led to a significant activity drop to 1779 h^-1^. The selectivity remained stable at an optimal level across this entire temperature series. It is important to note the fundamental difference in the calculation methods of TOF between the uncoated and coated catalysts. For the uncoated Pt/AC, the true TOF was calculated based on the actual number of exposed surface Pt sites determined by CO chemisorption. However, for the Pt@C/AC series, the highly dense carbon shell completely blocks CO molecules from chemisorbing onto the inner Pt core (as confirmed by the H2-TPR results). Consequently, determining the active surface Pt sites for the core-shell catalysts via CO chemisorption is practically unfeasible. Therefore, we adopted the apparent TOF based on the total moles of Pt for the Pt@C/AC series. Due to the different normalization standards, the absolute TOF values of Pt/AC and Pt@C/AC cannot be directly compared. The apparent TOF values of the Pt@C/AC series are utilized strictly to compare the intrinsic activity trends (the volcano trend) among the coated samples themselves.

**TABLE 2:** Dynamics parameters and TOF calculation results of Pt/AC and Pt@C/AC-T series catalysts.

Catalyst	Initial reaction rate r_0_ (mol·L^-1^·h^-1^)	R^2^	Number of active sites n_active_ (mol)	TOF (h^-1^)
Pt/AC	1.305	1	2.31 × 10^−6^ ^(a)^	28,247
Pt@C/AC-600	0.297	0.96	7.69 × 10^−6^ ^(b)^	1,931
Pt@C/AC-700	0.4128	0.93	7.69 × 10^−6^ ^(b)^	2,684
Pt@C/AC-800	0.54	0.94	7.69 × 10^−6^ ^(b)^	3,511
Pt@C/AC-900	0.2736	0.97	7.69 × 10^−6^ ^(b)^	1,779

Reaction conditions: 0.05 g catalyst, 0.5 g p-CNB, 50 mL methanol, P = 1 MPa, r = 1,000 rpm, T = 80 °C.

The TOF, value for Pt/AC, is the True TOF, normalized by exposed surface Pt sites. The TOF, values for the Pt@C/AC, series are Apparent TOFs, normalized by the total moles of Pt. These two types of TOF, values are fundamentally different in normalization and should not be directly compared numerically.

(a) True TOF, normalized by the number of exposed surface Pt sites (determined by CO chemisorption); (b) Apparent TOF, normalized by the total moles of Pt in the catalyst. These footnote symbols correspond to the values listed in the “Number of active sites” column.

This nonlinear activity variation reveals a complex structure-activity relationship. Further in-depth investigations are necessary. These subsequent studies will uncover the intrinsic mechanism behind this volcano trend.

**FIGURE 2:**
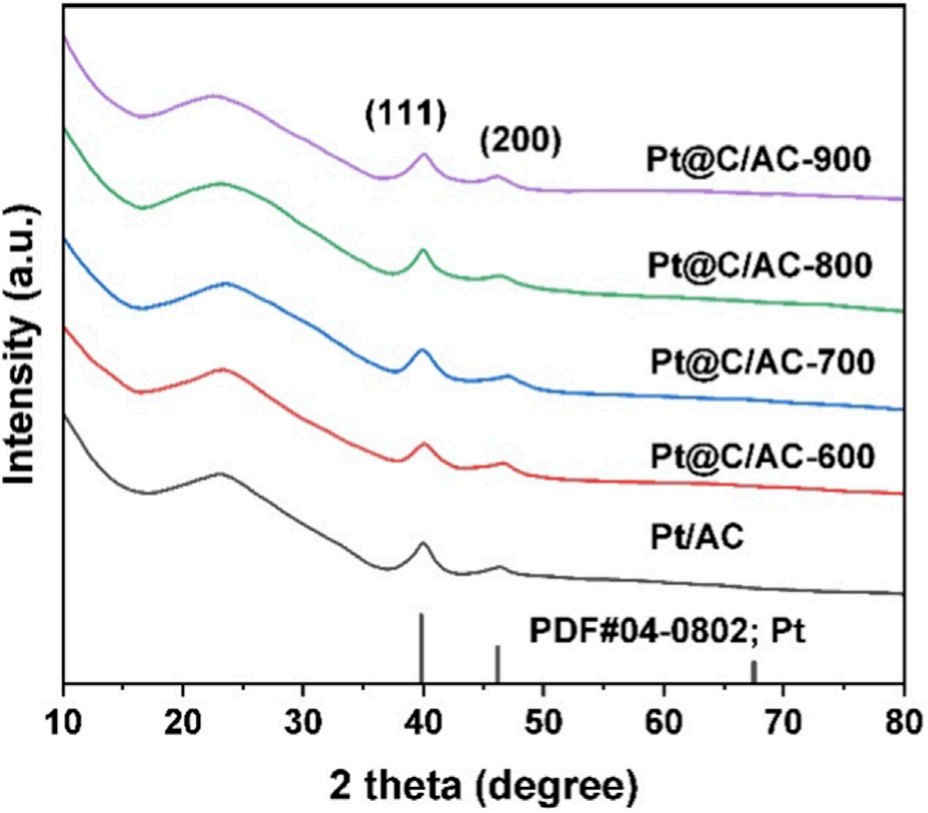
XRD patterns of each catalyst.

X-ray diffraction analysis was performed to determine the crystal structures of the samples. Figure 2 shows the X ray diffraction patterns of the samples. All catalysts show a broad peak near 24.5. belonging to the amorphous carbon carrier. The uncoated precursor displays clear signals of face-centered cubic platinum metal. The primary peak at 39.8 represents the (111) crystal plane of the fcc Pt (JCPDS No. 04-0802). The coated catalysts show identical metal diffraction patterns after the high-temperature pyrolysis. The crystal phase of the metal was fully preserved. Platinum crystallite sizes were calculated with the Scherrer equation. The initial metal size on the bare carrier is 3.4 nm. The sizes for all coated catalysts are maintained in a narrow range of 3.5–3.6 nm. The protective carbon layer effectively prevented metal sintering at elevated temperatures. The uniform metal particle size across all samples eliminates the size effect as a main reason for the volcano-type activity trend.

**FIGURE 3:**
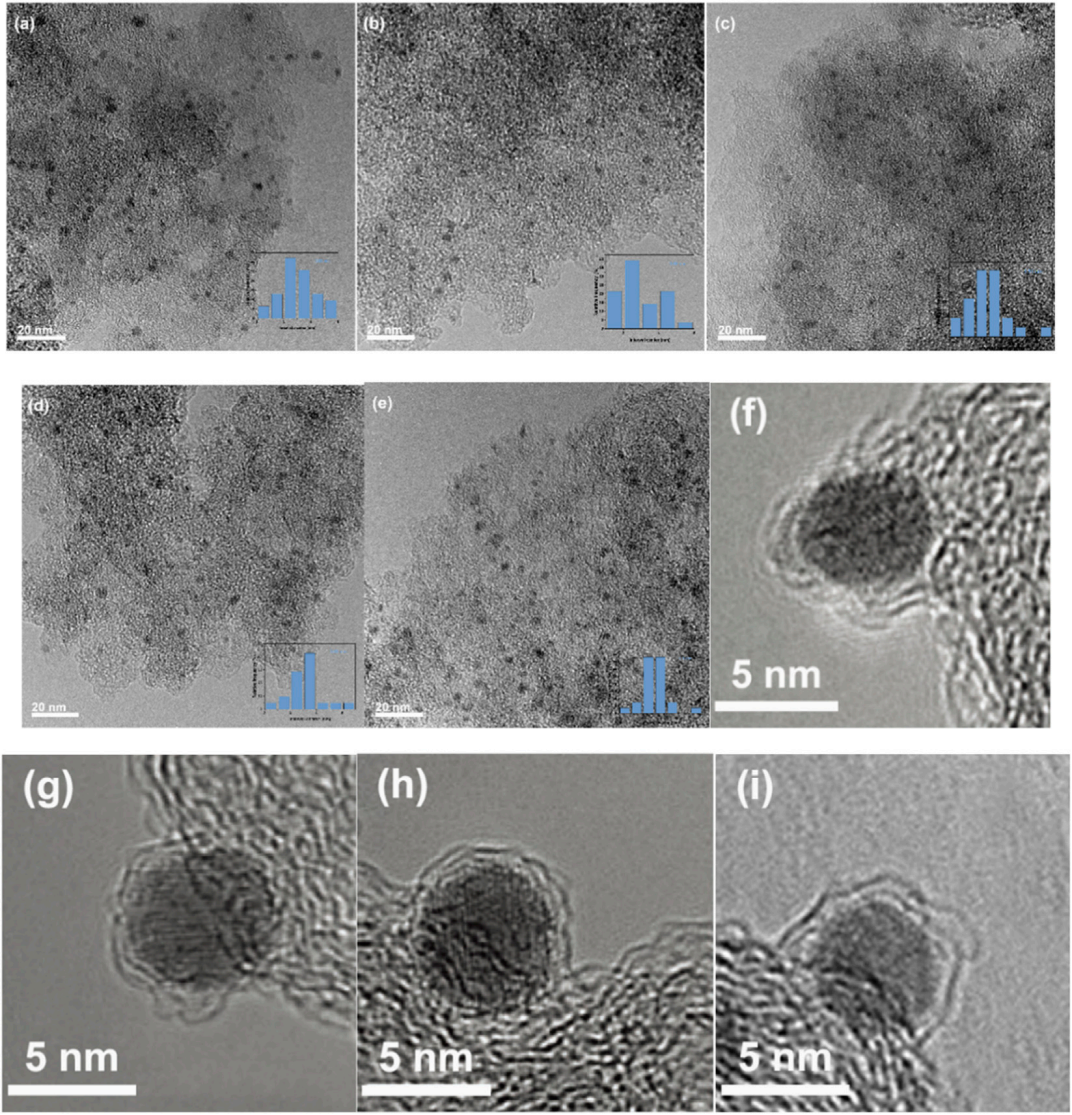
TEM images of **(a)** Pt/AC, **(b)** Pt@C/AC-600, **(c)** Pt@C/AC-700, **(d)** Pt@C/AC-800, **(e)** Pt@C/AC-900, with particle size distribution histograms inset; high-magnification TEM images of **(f)** Pt/AC, **(g)** Pt@C/AC-600, **(h)** Pt@C/AC-800, **(i)** Pt@C/AC-900.

Transmission electron microscopy was used to examine the morphology of the catalysts. Figure 3 presents the transmission electron microscopy images of the catalysts. For the uncoated precursor, dark platinum nanoparticles are uniformly dispersed on the activated carbon support. The average particle size is 3.53 nm. The coated sample maintains similar metal dispersion. The average platinum size of all pyrolyzed samples remains stable within 3.5–3.6 nm as measured by statistical measurements. The local structures are shown by high-magnification images. A light-colored, uniform carbon layer fully covers each dark platinum nanoparticle. This indicates that the core-shell structure has been successfully constructed. The uniform metal size and stable core-shell morphology are consistent with XRD results. This further confirms that the change in activity is not related to differences in geometric size.

**FIGURE 4:**
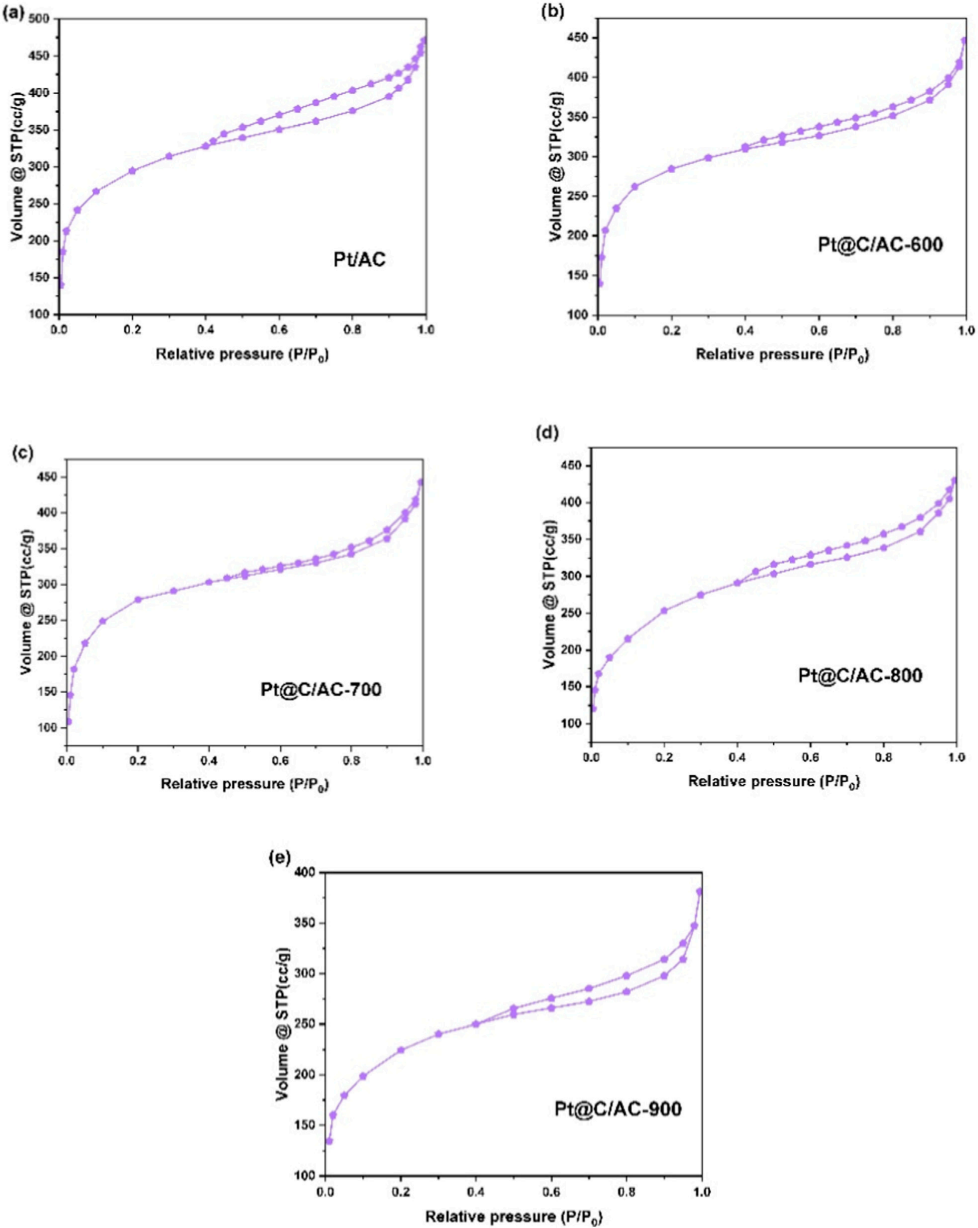
N2 adsorption-desorption isotherms of Pt/AC **(a)** and the Pt@/AC-T catalysts **(b–e)**.

The nitrogen adsorption-desorption tests were performed to characterize porous textures. Nitrogen adsorption-desorption isotherms are shown in Figure 4. The relevant data is listed in Table 3. All samples have type IV isotherms with hysteresis loops. The materials have mesoporous structures. Pt/AC has a specific surface area of 1,035 m^2^/g. The coated sample treated at 600 °C displays a reduced area of 958 m^2^/g. The generated carbon layer partially filled the original pores. The structural parameters remain stable across the entire coated series. The surface areas range from 945 to 958 m^2^/g. The pore volumes stay constant around 0.47–0.48 cm^3^/g. The high temperature pyrolysis process caused minimal changes to the macroscopic textures. All coated catalysts provide similar mass transfer channels. The observed volcano-type activity trend is unrelated to surface area variations.

**TABLE 3:** Texture parameters of Pt/AC and Pt@C/AC-T series catalysts.

Catalyst	S_BET_ (m^2^/g)	V_pore_ (cm^3^/g)	R_pore_ (nm)
Pt/AC	1,035	0.51	2.87
Pt@C/AC-600	958	0.48	2.91
Pt@C/AC-700	952	0.48	2.93
Pt@C/AC-800	948	0.47	2.95
Pt@C/AC-900	945	0.47	2.96

**FIGURE 5:**
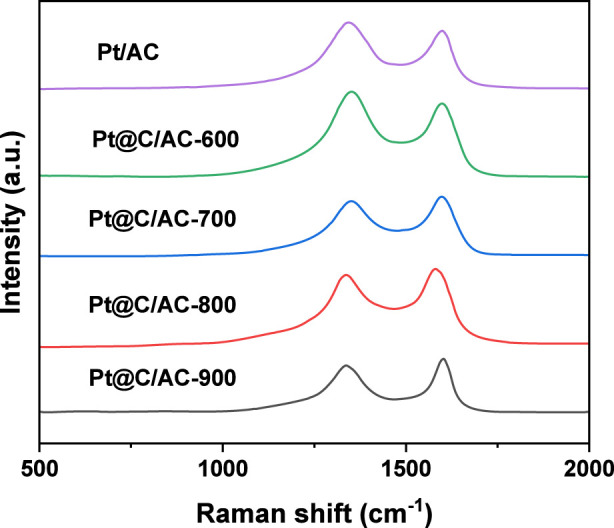
Raman spectra of each catalyst.

Raman spectroscopy was applied to analyze the structural order of the carbon materials. Figure 5 provides the corresponding profiles. All samples display typical D and G bands near 1,350 and 1,590 cm^-1^. The intensity ratio of D/G band represents the defect density (Yao et al., 2024b). The uncoated precursor has a ratio of 1.05. The sample pyrolyzed at 600 °C shows an increased ratio of 1.12. The newly formed carbon from glucose initially adds disordered structures. The ratio decreases continuously as the pyrolysis temperature rises. The value drops to 0.98 for the 700 °C sample. The 800 °C sample exhibits a ratio of 0.91. The lowest value of 0.85 is reached at 900 °C. The thermal treatment eliminates structural defects. The graphitization degree of the carbon shell increases systematically with the rising temperature.

**FIGURE 6:**
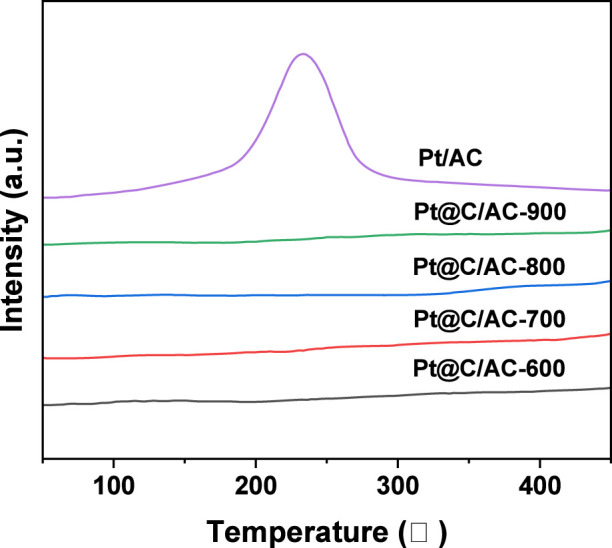
H_2_-TPR profiles of Pt/AC and the Pt@C/AC-T catalysts.

Hydrogen temperature programmed reduction (H_2_-TPR) was applied to evaluate the shell compactness. Figure 6 provides the H_2_-TPR profiles. The uncoated material shows an obvious hydrogen consumption peak near 232 °C. Surface platinum oxides are easily reduced on the exposed metal. All coated samples present flat lines across the entire tested temperature range. No reduction signals appear from 50 °C to 500 °C. The pyrolyzed carbon shell is highly dense. Hydrogen gas cannot penetrate this physical barrier to reach the inner Pt core. The catalytic hydrogenation has to occur on the exterior surface of the carbon shell.

**FIGURE 7:**
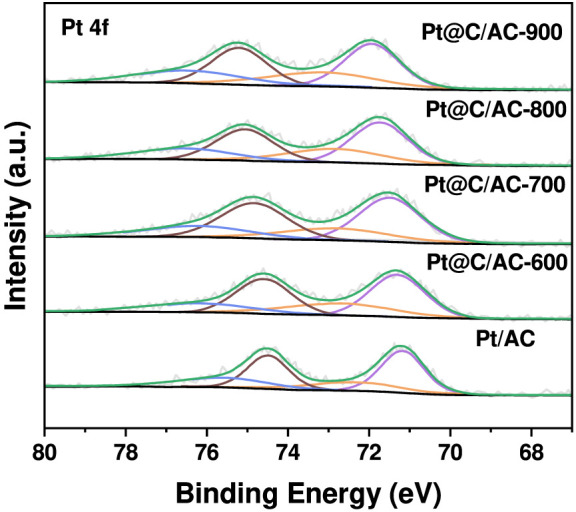
Pt 3d XPS spectra of Pt/AC and the Pt@C/AC-T catalysts.

X-ray photoelectron spectroscopy (XPS) was applied to analyze the surface chemical states. Figure 7 presents the spectra of Pt. Platinum 4f spectra were recorded. The zero-valent metal peak of the uncoated sample is located at 71.2 eV (Xie et al., 2025; Zhang et al., 2025). This peak of the sample coated at 600 °C shifts to 71.32 eV. The binding energy continues to shift positively with increasing pyrolysis temperature. The peak position of the 700 °C sample is at 71.51 eV, and the peak position of the 800 °C sample is at 71.73 eV. The largest value of 71.9 eV occurs when sample is coated at 900 °C. The systematic positive shift suggests a decreasing electron density of Pt. The inner Pt core transfers electrons to the outer carbon shell. A higher degree of graphitization results in a better conductive network. This structure change facilitates the interfacial electron transfer.

**FIGURE 8:**
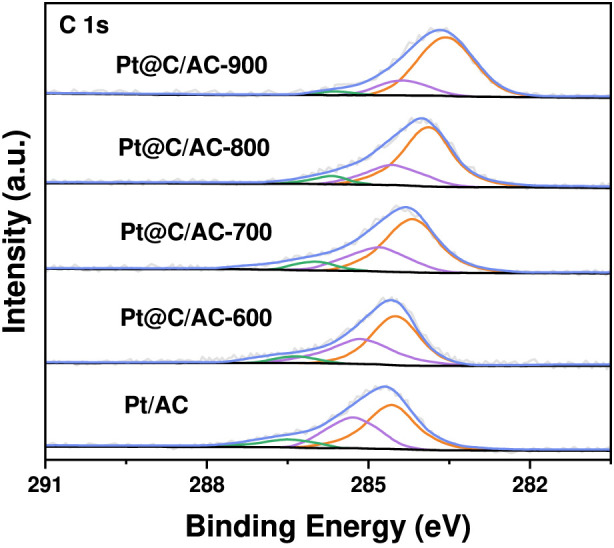
C 1s XPS spectra of Pt/AC and the Pt@C/AC-T catalysts.

The C 1s spectrum is used to investigate the composition of carbon shells. Figure 8 displays the Carbon 1s spectra. The main peak of sp^2^-hybridized carbon is located at 284.6 eV for the bare precursor (Yang et al., 2025). The binding energy shifts to lower values with increasing pyrolysis temperature. The sample treated at 600 °C shows a peak at 284.5 eV. The value drops to 283.91 eV at 800 °C. The 900 °C sample exhibits the lowest value of 283.62 eV. This negative shift reflects an increase in electron density on the carbon atoms. The carbon shell receives electrons from the metal core. The peak area ratio of sp^3^ to sp^2^ carbon was also calculated. The results are listed in Table 4. The ratio decreases from 0.42 to 0.23 as the temperature rises from 600 °C to 900 °C. The thermal treatment converts disordered structures into stable graphitic networks. This structural evolution agrees with the Raman analysis.

**TABLE 4:** C 1s XPS peak fitting data of Pt/AC and Pt@C/AC-T series catalysts.

Catalyst	Sp^2^ C=C binding energy (eV)	Area (sp^3^)/Area (sp^2^)	Content of sp^2^ C (%)
Pt/AC	284.6	0.45	69
Pt@C/AC-600	284.5	0.42	70.4
Pt@C/AC-700	284.23	0.35	74.1
Pt@C/AC-800	283.91	0.29	77.5
Pt@C/AC-900	283.62	0.23	81.3

**FIGURE 9:**
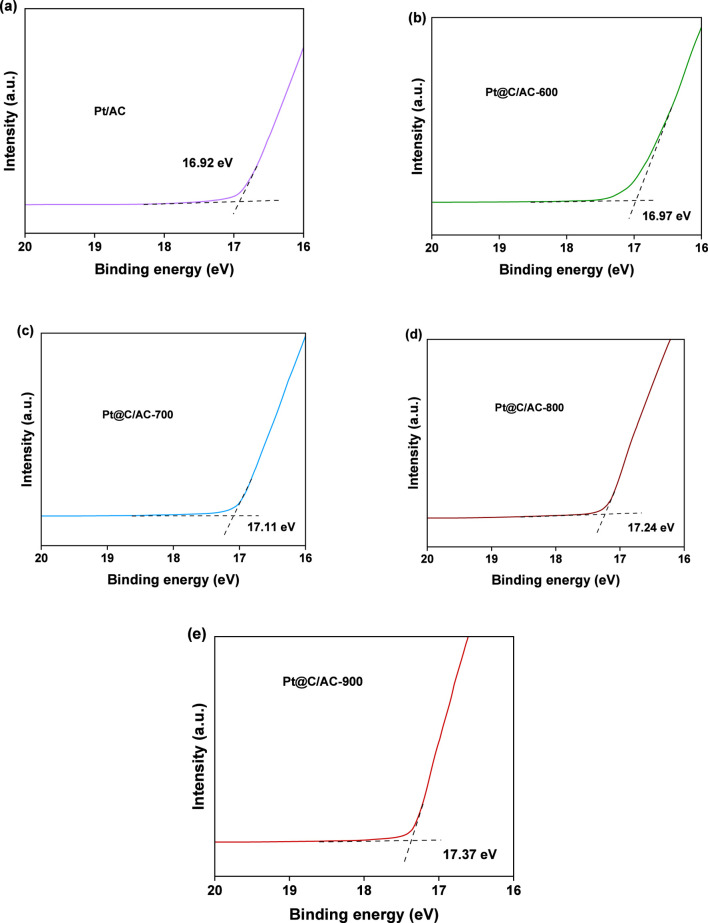
UPS secondary electron cut-off spectra of **(a)** Pt/AC, **(b)** Pt@C/AC-600, **(c)** Pt@C/AC-700, **(d)** Pt@C/AC-800, **(e)** Pt@C/AC-900.

**FIGURE 10:**
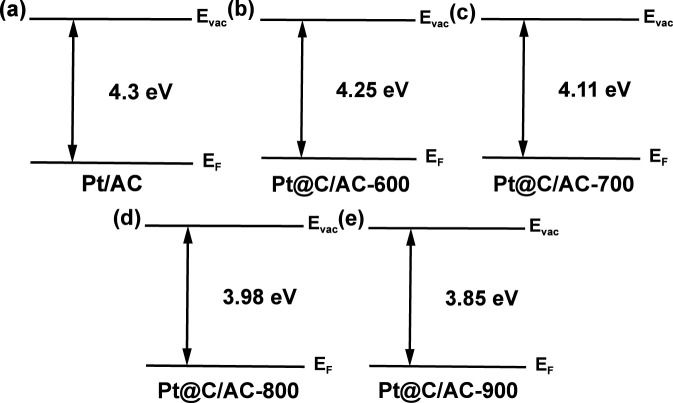
Schematic band structure diagrams of **(a)** Pt/AC, **(b)** Pt@C/AC-600, **(c)** Pt@C/AC-700, **(d)** Pt@C/AC-800, **(e)** Pt@C/AC-900.

Ultraviolet photoelectron spectroscopy was utilized to measure the work functions of the samples. Figure 9 shows the secondary electron cutoff regions. The uncoated precursor has a work function of 4.30 eV. The value drops to 4.25 eV for the coated sample pyrolyzed at 600 °C. The work function decreases continuously with higher pyrolysis temperatures. The 800 °C sample shows a value of 3.98 eV. The 900 °C sample exhibits the lowest value of 3.85 eV. Figure 10 illustrates the schematic band structures based on these measurements. The energy gap between the Fermi level and the vacuum level shrinks systematically at higher temperatures. A lower work function indicates a weaker electron-binding ability at the material surface. The carbon shell surface becomes increasingly electron-rich. This result agrees with those of XPS. The highly graphitized carbon network facilitates the electron acceptance from the Pt core and the delocalization of the electrons.

**FIGURE 11:**
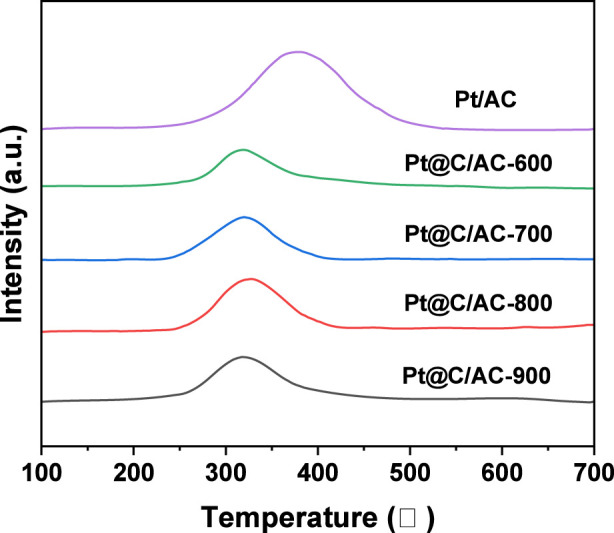
H_2_-TPD profiles of Pt/AC and Pt@C/AC-T catalysts.

Hydrogen temperature programmed desorption was used to study the hydrogen activation. Figure 11 shows the desorption profile. The Pt/C catalyst displays a large desorption peak at 380 °C. The bare Pt surface provides a large number of strong chemical adsorption sites. The area of desorption peak is much smaller for the coated series. The peak shifts towards the low-temperature zone at around 320 °C. The weaker chemical bond forms between the carbon shell and activated hydrogen species. The hydrogen desorption capacity of the encapsulation samples follows a volcanic trend. The 800 °C sample has the largest desorption peak area. The peak area and hydrogen activation capacity of the 900 °C sample have decreased. The optimum capacity of hydrogen adsorption and activation happens at 800 °C. This result is consistent with catalytic activity evaluation results.

**FIGURE 12:**
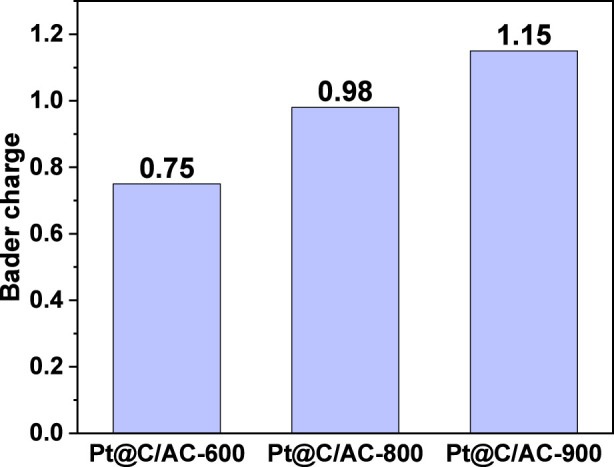
Bader charges of each catalyst.

Density functional theory calculations were applied to disclose the microscopic mechanisms. The three different core-shell models were built up. They describe the samples pyrolyzed at 600, 800, and 900 °C. They correspond to carbon shells with high, medium, and low defect densities, respectively. Figure 12 shows the Bader charge analysis. The Pt core bears a positive charge in all models. The carbon shell is negatively charged. As the degree of graphitization increases, the number of transferred electrons also increases. The Pt core transfers 0.75 electrons to the carbon shell in the highly defective model. The electron loss of the Pt core increases to 1.15 in the highly graphitized model. This confirms the electron pump effect from the metal to the carbon shell. The highly graphitized carbon network provides superior electrical conductivity. This structural feature facilitates the electron pump effect from the inner metal. The outer carbon surface becomes highly electron-rich. This theoretical calculation agrees with the experimental spectroscopic findings.

**FIGURE 13:**
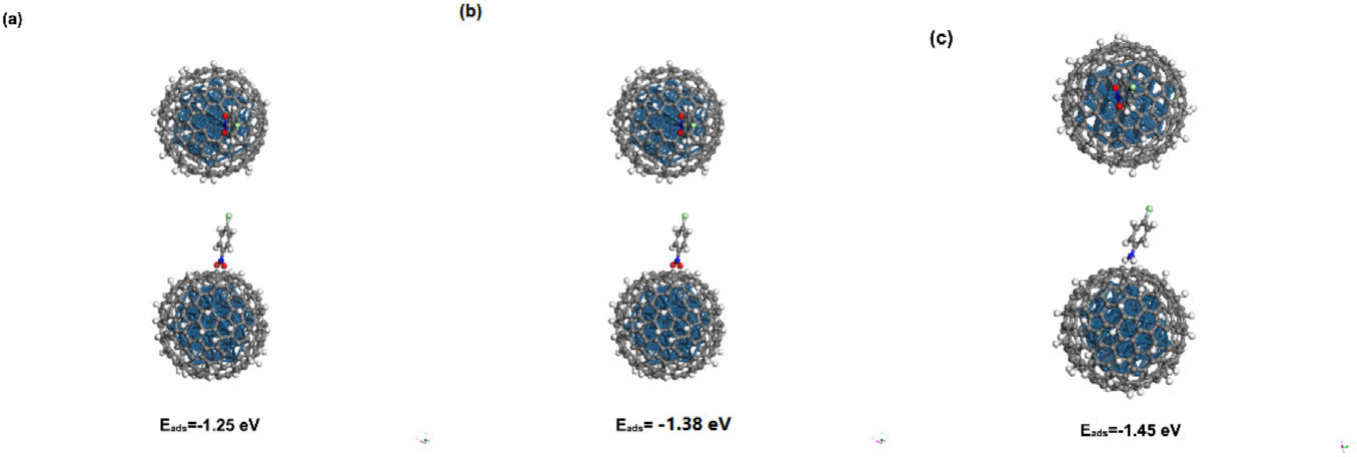
Adsorption configurations and energies of p-CNB on **(a)** Pt@C/AC-600, **(b)** Pt@C/AC-800, **(c)** Pt@C/AC-900 models.

Figure 13 displays the adsorption configurations and energies of the reactant p-chloronitrobenzene. The electron-deficient nitro group binds tightly to the electron-rich carbon surface. The adsorption energy on the 600 °C model is −1.25 eV. This value changes to −1.38 eV for the 800 °C model. The 900 °C model exhibits the strongest adsorption energy of −1.45 eV. The adsorption strength increases monotonically with the higher graphitization degree. A more electron-rich carbon surface provides better stabilization for the reactant molecule. This strong interaction provides a favorable thermodynamic condition to initiate the first hydrogenation step.

**FIGURE 14:**
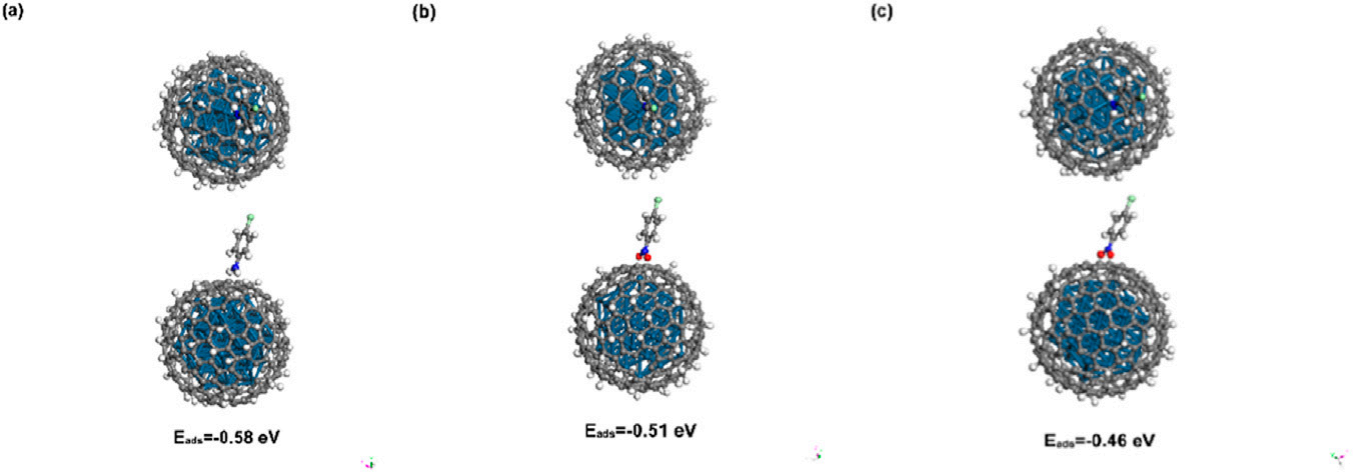
Adsorption configurations and energies of p-CAN on **(a)** Pt@C/AC-600, **(b)** Pt@C/AC-800, **(c)** Pt@C/AC-900 models.

Figure 14 shows the adsorption energies of the product p-chloroaniline. The product molecules show much weaker interactions with the carbon surface compared to the reactant. The adsorption energy is −0.58 eV on the 600 °C model. The value weakens to −0.51 eV for the 800 °C model. The 900 °C model shows the weakest adsorption energy of −0.46 eV. The amino group makes the product molecule relatively electron-rich. The highly graphitized carbon surface causes electrostatic repulsion. The generated product detaches rapidly from the catalyst. This rapid desorption prevents the over-hydrogenation side reaction. This thermodynamic behavior ensures the high catalytic selectivity.

**FIGURE 15:**
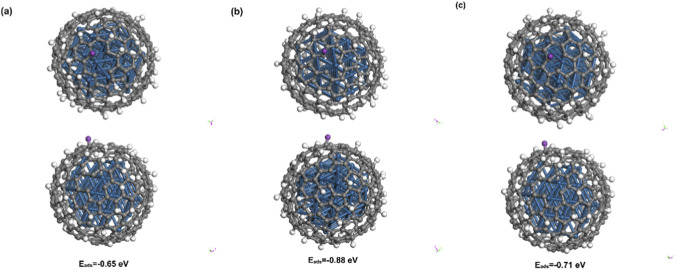
Adsorption configurations and energies of H atom on **(a)** Pt@C/AC-600, **(b)** Pt@C/AC-800, **(c)** Pt@C/AC-900 models.

The adsorption energy of a hydrogen atom was calculated to assess the thermodynamic stability of the samples. Figure 15 shows the calculation results. The adsorption strength shows a volcano-type trend. The 600 °C model generates the adsorption energy of −0.65 eV. The 800 °C model has the strongest adsorption energy of −0.88 eV. The adsorption energy weakens to −0.71 eV on the 900 °C model. This theoretical volcano trend is consistent with the experimental result of hydrogen desorption. Stronger adsorption strength represents a stronger ability to stabilize active hydrogen. The 800 °C model presents an ideal balance between surface electron enrichment and geometric defects. This kind of carbon surface provides the best anchor sites for hydrogen atoms.

**FIGURE 16:**
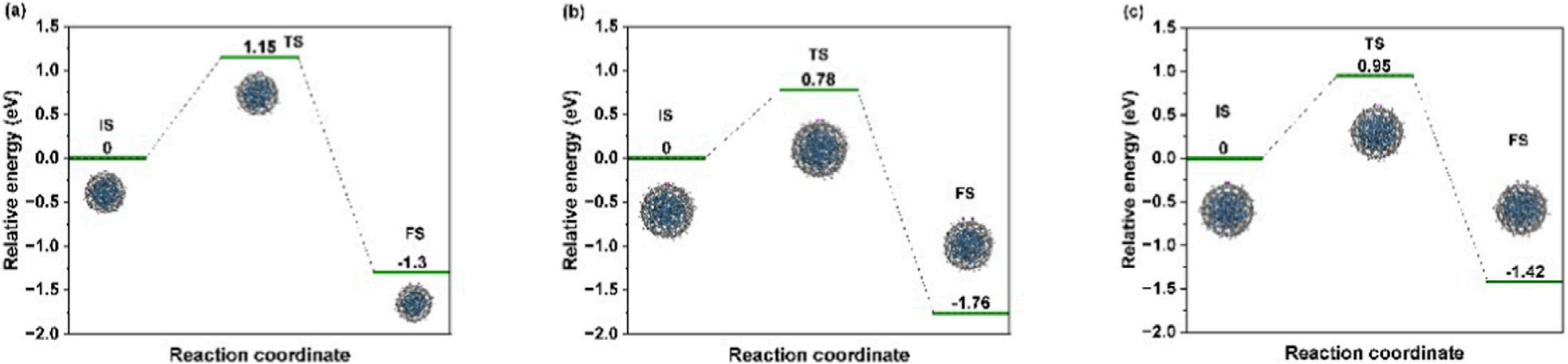
Hydrogen dissociation reaction pathways on **(a)** Pt@C/AC-600, **(b)** Pt@C/AC-800, **(c)** Pt@C/AC-900 models.

The potential energy surface of hydrogen molecule dissociation was computed to obtain the kinetic barriers. Figure 16 shows the reaction pathways. The dissociation barrier shows an anti-volcano-type trend. The 600 °C model generates the activation energy of 1.15 eV. The 800 °C model has the lowest kinetic barrier of 0.78 eV. It increases to 0.95 eV on the 900 °C model. This theoretical anti-volcano-type inversely mirrors the activity curve. The lowest activation barrier causes the highest catalytic activity directly. The 800 °C catalyst has the most favorable surface structure for H-H bond cleavage.

Based on the comprehensive experimental and theoretical results, the trend of the volcano-type activity can be elucidated. The properties of carbon shell depend on the synergistic effect of electronic and geometric effects. During the temperature range of 600 °C–800 °C, electronic effects play a leading role. The increase in graphitization degree promotes the electron transfer from Pt to the carbon shell. Specifically, the initial carbon shell derived from glucose inherently possesses a high density of defects, namely, sp^3^ carbon and vacancies. As the pyrolysis temperature increases, thermal annealing drives the rearrangement of the carbon skeleton, converting disordered structures into highly ordered sp^2^ graphitic lattices, which essentially acts as a defect-healing process. During the temperature range of 600 °C–800 °C, electronic effects play a leading role. This makes the carbon shell electron-rich. The enriched electrons reduce the hydrogen dissociation barrier. This electron enrichment reduces the hydrogen dissociation energy. The geometric effect is the limiting factor at 900 °C. The extensive thermal graphitization leads to an over-healing of the carbon lattice, causing the highly graphitized carbon shell to lose a large number of necessary surface defect sites. The highly graphitized carbon shell has lost a large number of necessary surface defect sites. The defects function as the required anchors for hydrogen adsorption and dissociation. Therefore, blindly increasing the pyrolysis temperature above 900 °Cwould lead to the complete elimination of these vital geometric defects, fundamentally preventing hydrogen activation and resulting in a further severe decline in catalytic activity. This inherently dictates why the sample treated at 800 °C achieves the optimal balance. The optimal catalyst pyrolyzed at 800 °C achieves a balance between high electron-density and sufficient geometric defects. The structural characteristics ensure the minimum kinetic barrier for the hydrogen activation.

**FIGURE 17:**
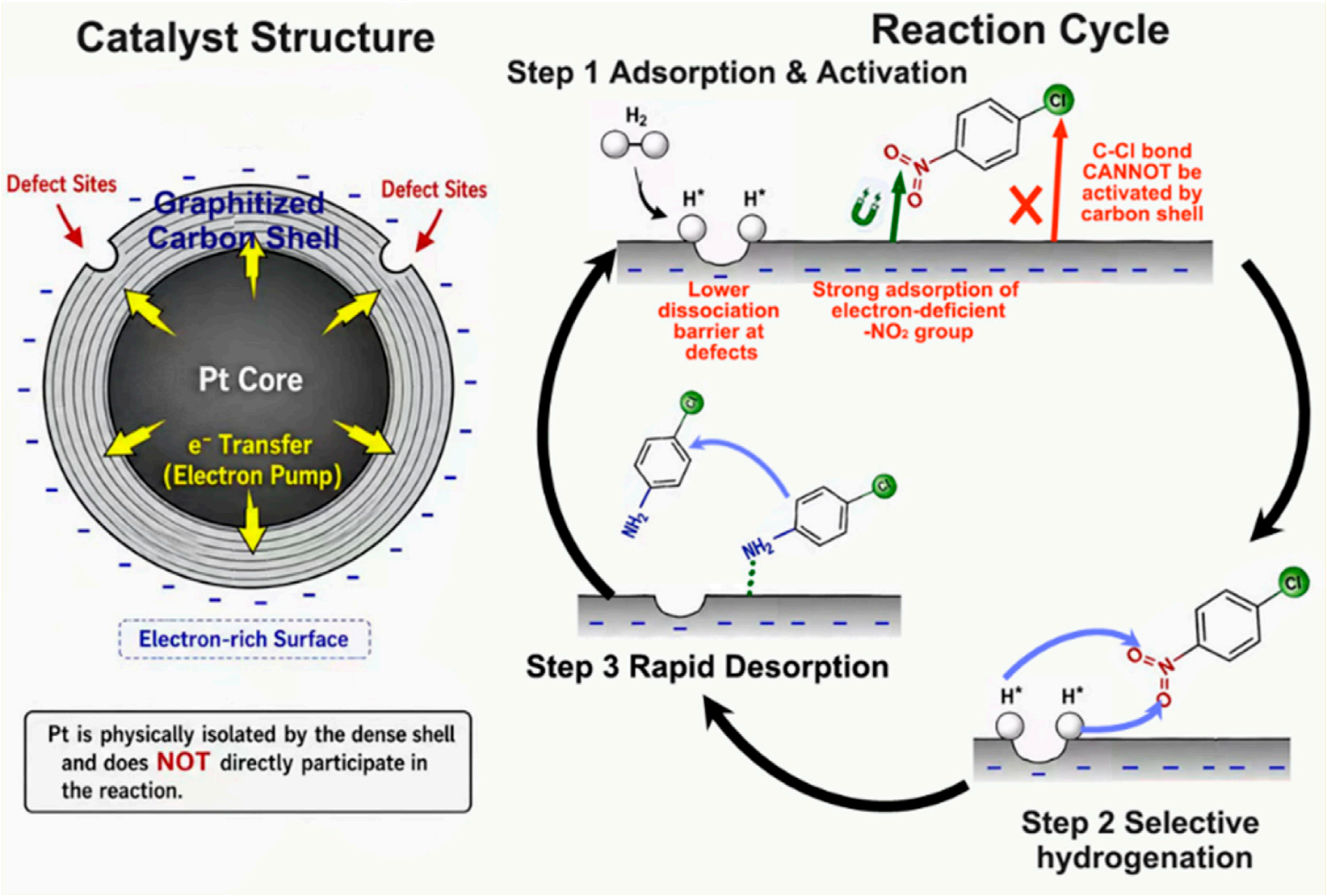
Schematic illustration of the catalytic mechanism over the Pt@C core-shell catalyst.

The high selectivity stems from the specific chemical nature of the carbon shell. The carbon material can not fulfill the activation of C-Cl bonds. The initial dechlorination pathway is blocked. Theoretical calculations confirm weak adsorption of products on carbon surfaces. The product quickly desorbs from the catalyst surface. The reactivity of activated hydrogen on the carbon surface is mild. That effectively suppresses the following over-hydrogenation side reactions. To summarize the entire process clearly, the true active sites in this system are the defect sites on the external surface of the electron-rich carbon shell, while the completely isolated Pt core does not directly participate in the reaction but acts strictly as an “electron pump”, continuously injecting electrons into the outer carbon shell to activate it, rather than just mechanically stabilizing it. The catalytic reaction proceeds as follows: First, H_2_ molecules anchor and dissociate at the residual geometric defect sites. Simultaneously, the electron-deficient nitro group of p-CNB strongly adsorbs onto the electron-rich carbon surface. Crucially, the carbon shell intrinsically cannot cleave C-Cl bonds, effectively avoiding initial hydrodechlorination. Finally, once hydrogenated, the resulting relatively electron-rich amino group of p-CAN experiences electrostatic repulsion from the carbon surface, leading to rapid desorption which prevents further over-hydrogenation. This holistic mechanism is visually illustrated in Figure 17.

Ten consecutive cycle tests were conducted to assess the catalyst stability. The performances of the optimal catalyst during the tests are summarized in Table 5. The conversion was maintained above 99.5% throughout the whole process. The selectivity of the target product is highly stable. The selectivity in the 10th cycle is 99.5%. The production of aniline by-products remains below 0.5%.

**TABLE 5:** Performance data of Pt@C/AC-800 catalyst after 10 cycles of application.

Run	p-CNB conversion (%)	Selectivity (%)	
		p-CAN	Aniline
1	100	99.8	0.2
2	100	99.8	0.2
3	100	99.7	0.3
4	100	99.7	0.3
5	100	99.9	0.1
6	100	99.6	0.4
7	100	99.4	0.6
8	100	99.7	0.3
9	100	99.8	0.2
10	100	99.5	0.5

**FIGURE 18:**
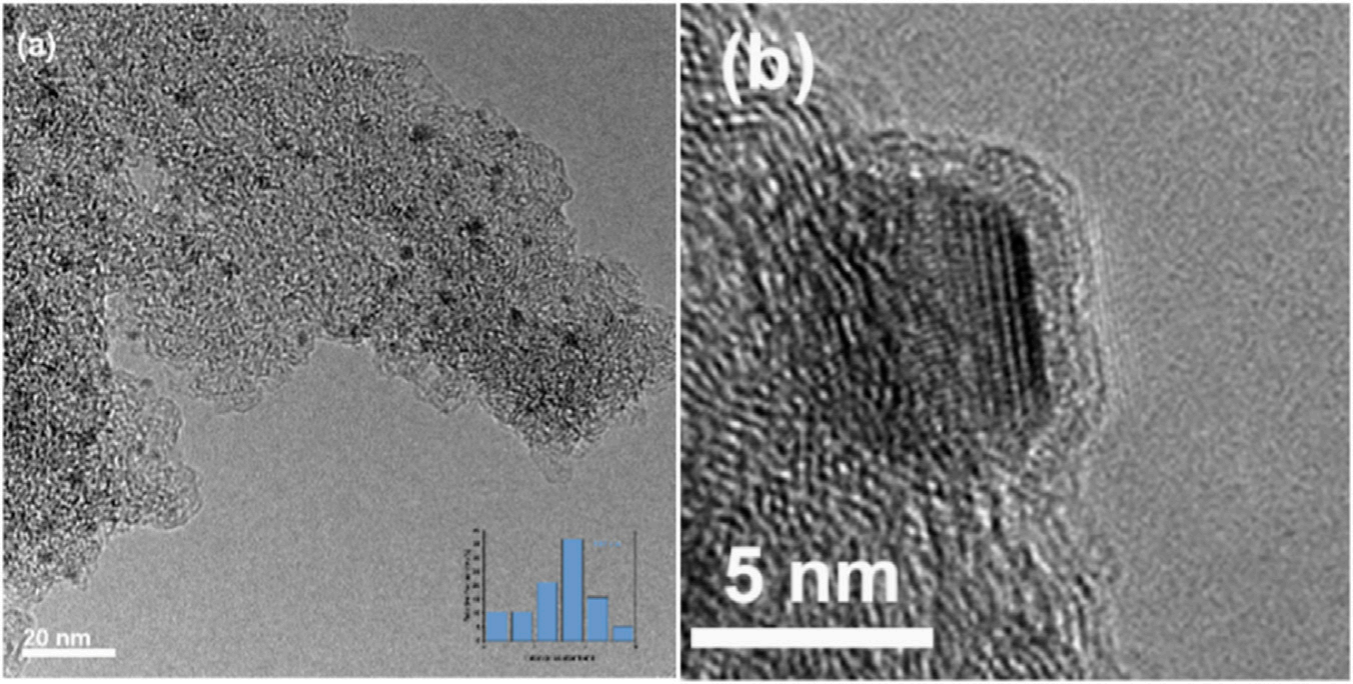
TEM image of Pt@C/AC-800-used catalyst: **(a)** Pt@C/AC-800-used low magnification panoramic image, **(b)** Pt@C/AC-800-used high-resolution image.

The used catalyst was characterized by transmission electron microscopy. The morphology of the sample after ten reaction cycles is revealed in Figure 18. The Pt nanoparticles remain uniformly dispersed on the support. The average particle size was measured to be 3.51 nm. No aggregation of Pt was observed. The high-magnification images reveal that the core-shell structure is well preserved. The protective carbon layer does not show any signs of damage. The sturdy carbon shell effectively restricts the Pt core. This physical barrier effectively avoids Pt sintering and affords excellent long-term stability. While this 10-cycle batch test serves as a preliminary laboratory-scale demonstration rather than a full commercial longevity evaluation, it strongly indicates the robust initial stability of the core-shell structure. Furthermore, since the optimal carbon shell was graphitized and structurally fixed at a high temperature of 800 °C, its graphitization degree and defect density remain thermodynamically highly stable under the mild liquid-phase reaction conditions at 80 °C. Future studies focusing on continuous-flow scale-up will further track the long-term structural evolution of these carbon interfaces under industrial conditions.

## 4 Conclusion

In this study, a series of supported core-shell catalysts with controllable graphitization degree were successfully synthesized. They presented excellent selectivity toward the selective hydrogenation. The carbon shell does not possess the ability to activate C-Cl bonds and the product can quickly desorb from the surface. The catalytic activity exhibits a volcano-type trend based on the pyrolysis temperature. The sample pyrolyzed at 800 °C provided the highest turnover frequency. The experimental and theoretical results indicate an electronic pump effect from the Pt core to the external carbon shell. The optimal activity stems from the balance between electron enrichment of the carbon shell and geometric defects. This specific structural balance can result in the minimum kinetic barrier for hydrogen dissociation. The sturdy carbon shell also affords excellent long-term stability against Pt aggregation. This work provides a feasible strategy for designing efficient and stable carbon-encapsulated catalysts.

## Data Availability

The raw data supporting the conclusions of this article will be made available by the authors, without undue reservation.
